# The PKA-CREB1 axis regulates coronavirus proliferation by viral helicase nsp13 association

**DOI:** 10.1128/jvi.01565-23

**Published:** 2024-03-06

**Authors:** Tong Zheng, Beilei Shen, Yu Bai, Entao Li, Xun Zhang, Yong Hu, Ting Gao, Qincai Dong, Lin Zhu, Rui Jin, Hui Shi, Hainan Liu, Yuwei Gao, Xuan Liu, Cheng Cao

**Affiliations:** 1 Genetic Engineering Research Laboratory, Beijing Institute of Biotechnology, Beijing, China; 2 Changchun Veterinary Research Institute, Chinese Academy of Agricultural Sciences, Changchun, China; 3 Institute of Physical Science and Information Technology, Anhui University, Hefei, Anhui, China; 4 Division of Life Sciences and Medicine, University of Science and Technology of China, Hefei, Anhui, China; The Ohio State University, Columbus, Ohio, USA

**Keywords:** nsp13, CREB1, helicase, SARS-CoV-2, viral replication

## Abstract

The COVID-19 pandemic caused by severe acute respiratory syndrome coronavirus 2 (SARS-CoV-2) has posed a worldwide threat in the past 3 years. Although it has been widely and intensively investigated, the mechanism underlying the coronavirus-host interaction requires further elucidation, which may contribute to the development of new antiviral strategies. Here, we demonstrated that the host cAMP-responsive element-binding protein (CREB1) interacts with the non-structural protein 13 (nsp13) of SARS-CoV-2, a conserved helicase for coronavirus replication, both in cells and in lung tissues subjected to SARS-CoV-2 infection. The ATPase and helicase activity of viral nsp13 were shown to be potentiated by CREB1 association, as well as by Protein kinase A (PKA)-mediated CREB1 activation. SARS-CoV-2 replication is significantly suppressed by PKA Cα, cAMP-activated protein kinase catalytic subunit alpha (PRKACA), and CREB1 knockdown or inhibition. Consistently, the CREB1 inhibitor 666-15 has shown significant antiviral effects against both the WIV04 strain and the Omicron strain of the SARS-CoV-2. Our findings indicate that the PKA-CREB1 signaling axis may serve as a novel therapeutic target against coronavirus infection.

**IMPORTANCE:**

In this study, we provide solid evidence that host transcription factor cAMP-responsive element-binding protein (CREB1) interacts directly with severe acute respiratory syndrome coronavirus 2 (SARS-CoV-2) helicase non-structural protein 13 (nsp13) and potentiate its ATPase and helicase activity. And by live SARS-CoV-2 virus infection, the inhibition of CREB1 dramatically impairs SARS-CoV-2 replication *in vivo*. Notably, the IC50 of CREB1 inhibitor 666-15 is comparable to that of remdesivir. These results may extend to all highly pathogenic coronaviruses due to the conserved nsp13 sequences in the virus.

## INTRODUCTION

Coronaviruses (CoVs) cause respiratory and intestinal infections in animals and humans (1). The newly identified severe acute respiratory syndrome coronavirus 2 (SARS-CoV-2) has resulted in a global health emergency because of its rapid spread, leading to >767 million infections and >6 million related deaths as of 12 July 2023 (https://covid19.who.int/). While vaccines and antiviral drugs to control the disease have been successfully developed, the detailed mechanism underlying virus-host interactions requires further investigation.

CoVs are enveloped viruses with a positive-sense single-stranded RNA genome of approximately 30 kb (2). The CoV genome encodes four structural proteins: the spike protein (S), envelope protein (E), membrane glycoprotein (M), and nucleocapsid protein (N) (3, 4). The 5′-terminal two-thirds of the genome encodes two large precursors (ORF1a and ORF1b), which are further cleaved into 16 non-structural proteins that are involved in RNA replication and transcription (5). Non-structural protein 13 (nsp13), a superfamily 1B (SF1B) helicase encoded by ORF1b, plays an important role in the CoV replication-transcription complex by unwinding double-stranded nucleic acid helices into single-stranded nucleic acids (6, 7). Nsp13 is highly conserved among CoVs and shares 99.8% identity in the primary amino acid sequences between SARS-CoV and SARS-CoV-2 (8). It has been reported that the viral helicase nsp13 forms a stable complex with viral holo-RdRp (RNA-dependent RNA polymerase holoenzyme) during viral replication and transcription (6). Regarding viral-host interactions, nsp13 has been reported to suppress RNA virus-induced type I interferon production (9, 10). Nsp13 interacts with host TBK1, thereby suppressing TBK1-mediated IRF3 phosphorylation. It has also been established that nsp13 exploits the host deubiquitinase USP13 to stabilize itself (10).

Protein kinase A (PKA) is a cAMP-dependent serine/threonine kinase that senses stress signals such as extracellular hormones (11, 12). Its catalytic subunit, cAMP-activated protein kinase catalytic subunit alpha (PRKACA), phosphorylates a series of downstream substrates including cAMP-responsive element-binding protein (CREB1). It can regulate cellular signal transduction (such as cell growth, metabolism, and immunity) by regulating the transcription of various target genes through CREB1 phosphorylation at serine residue 133 (12, 13). PKA signaling is also involved in Ebola virus, HIV-1, hepatitis B virus, Zika virus, and adenovirus infection (14–18). However, its potential role in CoV infection and pathogenesis remains unclear. In an early study of the SARS-CoV-2 interactome, PRKACA was detected in anti-SARS-CoV-2 helicase (nsp13) immunoprecipitates by affinity purification-mass spectrometry analysis, suggesting that nsp13 may interact with host PRKACA (19). A more detailed investigation should be performed to elucidate the exact role of the host PKA signaling pathway during SARS-CoV-2 infection.

In this study, the SARS-CoV-2 helicase nsp13 was demonstrated to interact with PKA and CREB1, and SARS-CoV-2 replication was significantly facilitated by PKA-CREB1 activation. The discovery of the crucial roles of PKA-CREB1 signaling in viral replication provides a new approach to the development of broad-spectrum CoV therapies.

## RESULTS

### SARS-CoV-2 helicase nsp13 interacts with host PRKACA and CREB1

**Fig 1:**
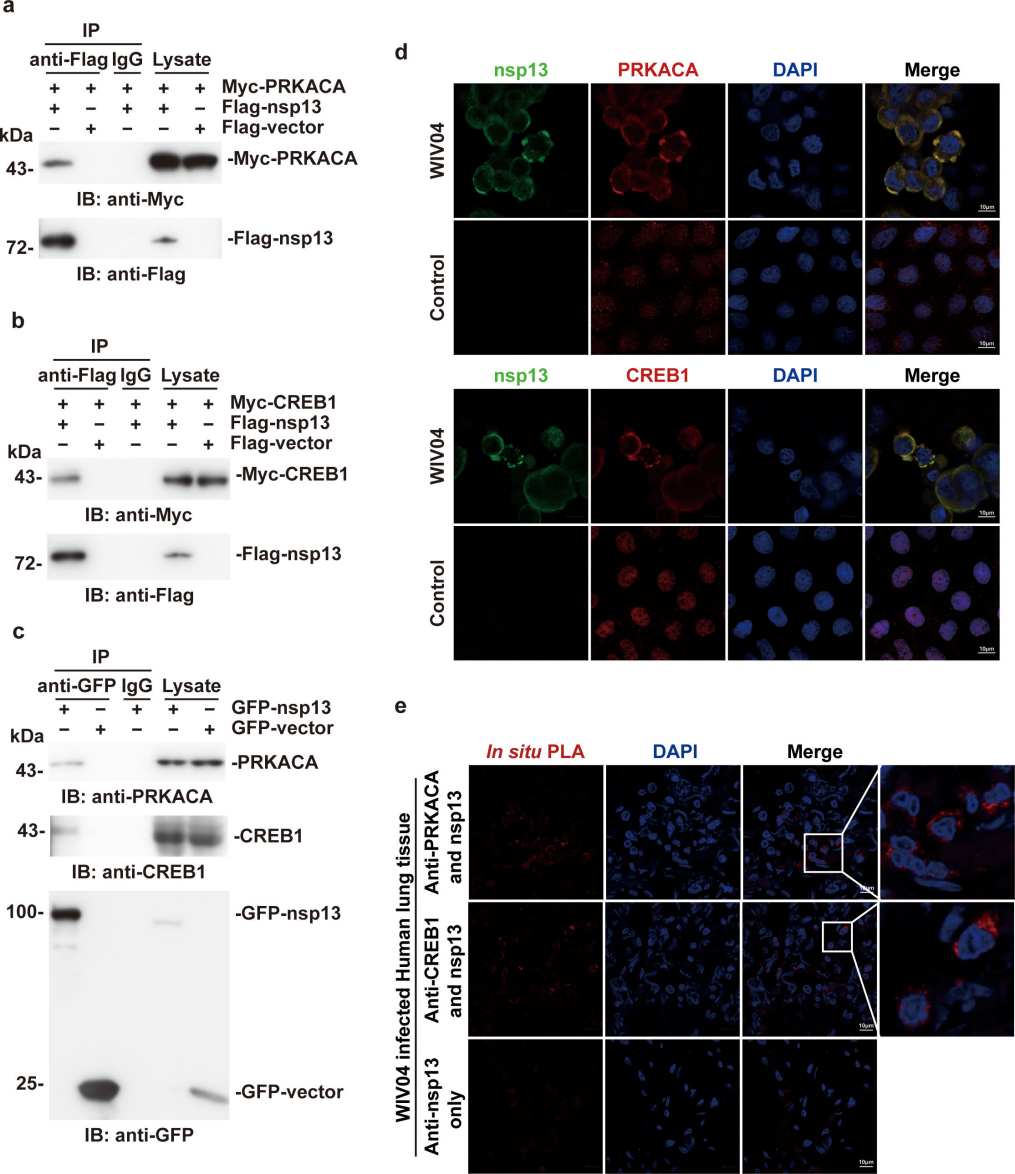
nsp13 interacts with host PRKACA and CREB. (a) Lysates of HEK293T cells expressing Myc-PRKACA and Flag-nsp13 or Flag-vector were subjected to immunoprecipitation with an anti-Flag antibody and analyzed by immunoblotting. (b) Lysates of HEK293T cells expressing Myc-CREB1 and Flag-nsp13 or Flag-vector were subjected to immunoprecipitation with an anti-Flag antibody and analyzed by immunoblotting. (c) Lysates of HEK293T cells transfected with GFP-nsp13 or GFP-vector were subjected to immunoprecipitation with an anti-GFP antibody, and the immunoprecipitates were analyzed by immunoblotting with anti-CREB1 and anti-PRKACA antibodies. (d) Caco-2 cells infected with SARS-CoV-2 [WIV04; multiplicity of infection (MOI) = 10; upper panel] or untreated cells (lower panel) were fixed at 48 hpi and immunolabeled with anti-nsp13 (green) and anti-PRKACA or anti-CREB1 (red) antibodies. (**e)** Lung tissue sections of patients infected with 2019BetaCoV (WIV04) were subjected to an *in situ* proximity ligation assay (PLA) with anti-nsp13 and anti-PRKACA or anti-CREB1 antibodies. At least three independent replicates of each experiment were performed.

Affinity purification-mass spectrometry analysis indicated that the SARS-CoV-2 helicase nsp13 associates with host PRKACA (19). To confirm previous screening results, Flag-tagged nsp13 and Myc-tagged PRKACA were coexpressed in 293T cells, and Myc-PRKACA was detected in the Flag-nsp13 immunoprecipitates but not in the Flag-vector immunoprecipitates (Fig. 1a). Moreover, the transcription factor CREB1, a downstream substrate of PKA, was also coimmunoprecipitated with Flag-nsp13 (Fig. 1b). In concert with the overexpression results, the association of endogenous PRKACA and CREB1 with GFP-nsp13 was also observed by immunoprecipitation (Fig. 1c), indicating that viral nsp13 could form complexes with PRKACA and CREB1 in host cells.

To further investigate the colocalization of viral nsp13 with host factors, A549 cells were transfected with Flag-nsp13 and immunostained with anti-nsp13 and anti-PRKACA or anti-CREB1 antibodies. The results showed that Flag-nsp13 colocalized with endogenous PRKACA and CREB1 in the cytoplasm (Fig. S1a and b). As expected, the colocalization of nsp13 with PRKACA and CREB1 was also observed in SARS-CoV-2 (2019BetaCoV/WIV04)-infected Caco-2 cells, which are highly permissive for SARS-CoV-2 infection (Fig. 1d). Notably, the cellular distribution pattern of CREB1 was significantly changed to the cytoplasm by infection.

Next, an *in situ* Duolink proximity ligation assay (PLA) with high specificity and sensitivity was performed in SARS-CoV-2-infected lung tissue. Obvious nsp13:PRKACA and nsp13:CREB1 association signals (shown in red) were observed in COVID-19 autopsy lung tissues (Fig. 1e), as well as in mouse-adapted SARS-CoV-2-infected mouse lung tissues (Fig. S1c). These results collectively demonstrated that nsp13 of SARS-CoV-2 interacts with PRKACA and CREB1 in SARS-CoV-2-infected cells.

### CREB1 interacts directly with the nsp13 2a domain

**Fig 2:**
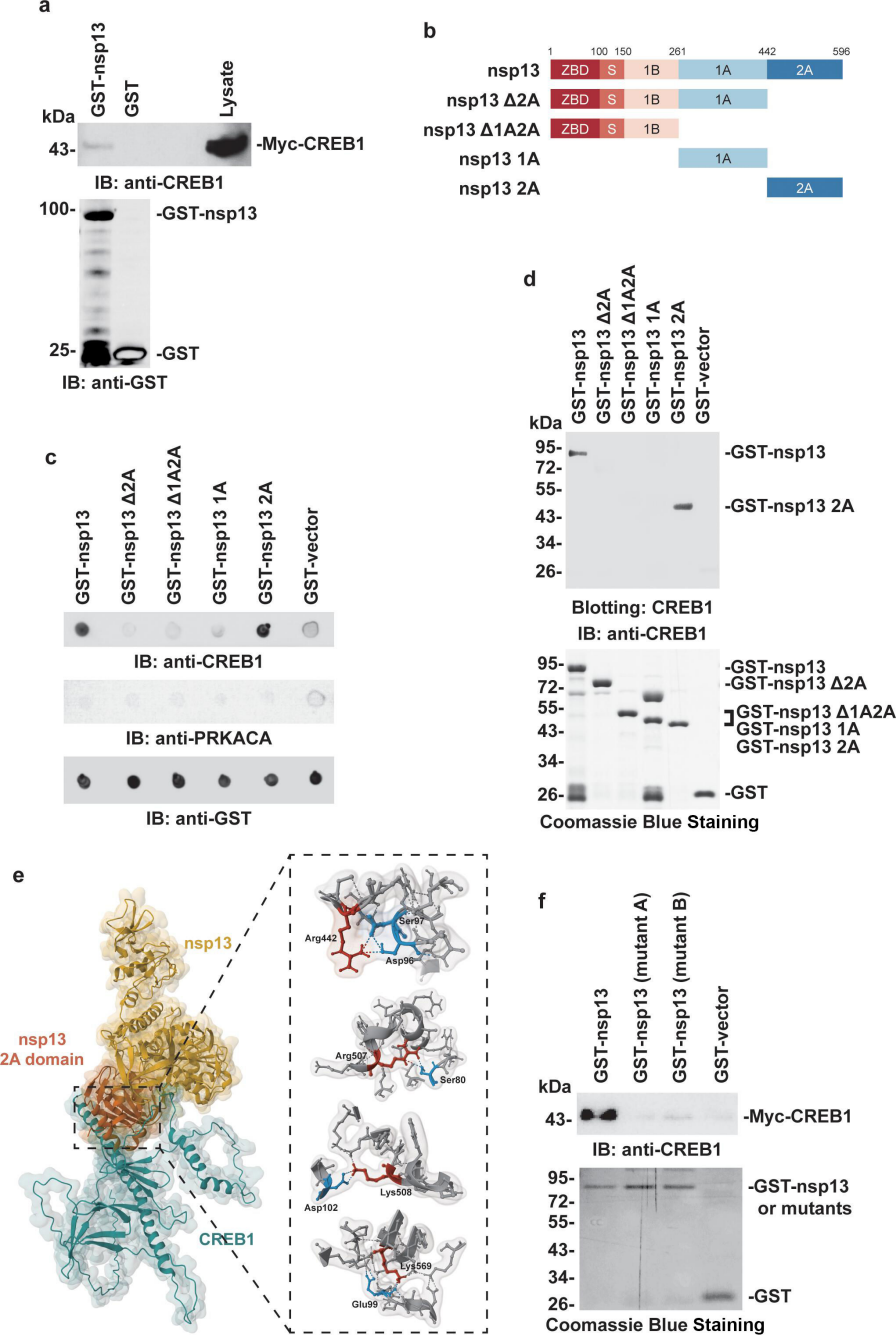
CREB1 interacts directly with the nsp13 2A domain. (a) The interaction of SARS-CoV-2 nsp13 with CREB1 was investigated by a GST pulldown assay. Lysates of HEK293T cells transfected with the Myc-CREB1 plasmid were subjected to precipitation with GST-nsp13 and GST agarose beads. Precipitates and lysates were analyzed by immunoblotting, and GST protein was used as a negative control. (b) Schematic representation of nsp13 domains. Nsp13 is composed of a zinc-binding domain (red), stalk domain (orange), 1B domain (light pink), 1A domain (light blue), and 2A domain (blue). (**c)** Purified GST-nsp13 and its mutants were dotted onto nitrocellulose membrane and then incubated with purified CREB1 or PRKACA. The direct binding of the GST fusion proteins was detected using an anti-CREB1 or anti-PRKACA antibody and GST protein was used as a negative control (anti-GST indicates equal sample loading). (**d)** Purified GST-nsp13 and its mutants [described in (b)] were resolved by SDS-PAGE and transferred onto a polyvinylidene fluoride (PVDF) membrane. The PVDF membrane was incubated with purified CREB1 and then subjected to immunoblotting with an anti-CREB1 antibody. GST protein was used as a negative control. The purity of GST-nsp13 and its mutants were analyzed by Coomassie blue staining.** (e)** (Left) Three-dimensional modeling structure of the interaction between SARS-CoV-2 nsp13 (PDB ID: 7NIO; yellow) and CREB1 (predicted by Alpha Fold; cyan); the interaction domain of nsp13 is highlighted (orange). (Right) The possible binding site was simulated *in silico*; the binding residues of nsp13 are highlighted in red, and CREB1 is shown in cyan. The structural model of the nsp13-CREB1 complex is presented as a cartoon superimposed with a transparent molecular Gaussian surface. Images were created using Mol* at the PDB web app (20). (**f)** The interaction of SARS-CoV-2 nsp13 and its mutants with CREB1 was investigated by a GST pulldown assay. Lysates of HEK293T cells transfected with the Myc-CREB1 plasmid were subjected to precipitation with GST-nsp13, GST-nsp13 (mutant A), GST-nsp13 (mutant B), and GST agarose beads. Precipitates were analyzed by immunoblotting, and GST protein was used as a negative control. The purity of GST-nsp13 and its mutants were analyzed by Coomassie blue staining.

The interaction of SARS-CoV-2 nsp13 with host PRKACA or CREB1 was further investigated in detail. Prokaryotically expressed GST-nsp13 was constructed and purified in the *Escherichia coli* expression system (Fig. S2a). Then, lysates of cells expressing Flag-PRKACA or Myc-CREB1 were incubated with GST-nsp13 or GST-conjugated agarose beads. The results showed that Myc-CREB1 but not Flag-PRKACA was pulled down together with GST-nsp13. The GST adsorbates were used as a control (Fig. 2a; Fig. S2b). To further validate the direct association of nsp13 with CREB1, purified GST-nsp13 and CREB1 were subjected to dot blotting assay and far western blotting. Subsequent immunoblotting with an anti-CREB1 antibody demonstrated that nsp13 could interact with CREB1 directly *in vitro* (Fig. 2c and d). As controls, no such binding was observed when GST-nsp13 was incubated with purified PRKACA (Fig. 2c; Fig. S2c). These results supported a direct interaction between SARS-CoV-2 nsp13 and CREB1, which may subsequently mediate an indirect association of nsp13 with PRKACA, as we observed in previous immunoprecipitation and colocalization experiments.

Next, recombinant GST-tagged nsp13 truncation mutants were constructed and purified from *E. coli* (Fig. 2b) and subjected to dot blotting assay and far western blotting. The “RecA-like” helicase subdomain 2A, which is critical for nsp13 helicase activity and is responsible for nucleotide binding and hydrolysis, was observed to interact directly with CREB1 (Fig. 2c and d) (7). As controls, no CREB1 binding was observed with the GST-nsp13 1A domain or GST only. Accordingly, either nsp13 Δ2A or nsp13 Δ1A2A, in which the 2A domain was deleted, failed to interact with CREB1.

Furthermore, the interaction of CREB1 with nsp13 was assessed by *in silico* analysis, and the molecular docking model of SARS-CoV-2 nsp13 [from the X-ray diffraction data, PDB ID: 7NIO (21)] and CREB1 (predicted by Alpha Fold) generated using the ClusPro 2.0 web server (22, 23) (Fig. 2e, left) suggested that Arg442, Lys569, Arg507, and Lys508 in subdomain 2A of nsp13 are involved in the nsp13:CREB1 interaction (Fig. 2e, right). Correspondingly, when the Arg442, Arg507, Lys508, and Lys569 within the 2A subdomain were replaced by alanine or negative charged amino acid (Fig. S2d), the mutated nsp13 could barely bind CREB1 (Fig. 2f). These data collectively suggested that CREB1 interacted with viral nsp13 directly via subdomain 2A.

### Host CREB1 potentiates the ATPase and helicase activity of SARS-CoV-2 nsp13

**Fig 3:**
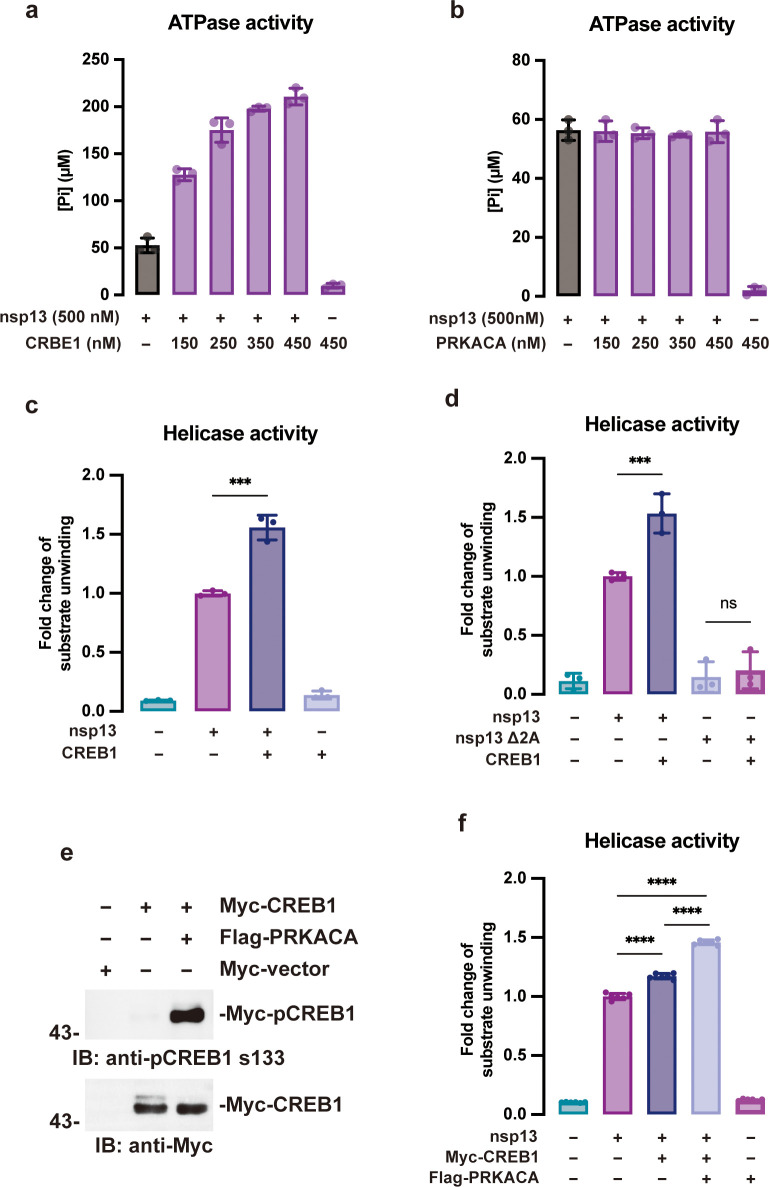
Host CREB1 promotes the ATPase and helicase activity of SARS-CoV-2 nsp13. (**a, b**) Recombinant GST-nsp13 (500 nM) was incubated with CREB1 or PKA at the indicated concentrations. The ATPase activity was measured based on the released free phosphate by a sensitive colorimetric assay. A reaction without nsp13 was used as a negative control. (**c)** Purified GST-nsp13 (100 nM) with or without CREB1 (50 nM) was incubated with 750 nM DNA substrate and 2 mM ATP at 25°C for 20 min, and the Cy3 signal was then measured at an excitation wavelength of 550 nm and an emission wavelength of 570 nm (a.u. = arbitrary units). A reaction containing the DNA substrate and ATP was used as a negative control.** (d**) The experiment was performed as described in (c), and the nsp13 Δ2A mutant (aa 1-442) was added instead of nsp13 as indicated. (e) Myc-CREB1 and s133-phosphorylated Myc-CREB1 were purified by a eukaryotic expression system (293T cells), and the phosphorylation level of purified proteins was analyzed by western blotting. (**f)** Purified GST-nsp13 (100 nM) and eukaryotic CREB1 (50 nM) were incubated with 750 nM DNA substrate and 2 mM ATP at room temperature for 20 min, and the Cy3 signal was measured as described in c. At least three independent replicates of each experiment were performed. Data are presented as the mean ± SEM (*P < 0.05, **P < 0.01, ****P < 0.0001).

Nsp13 is a helicase that also exhibits ATPase activity. To assess the effect of PKA/CREB1 on the biological function of nsp13, the ATPase and helicase activities of purified recombinant nsp13 were analyzed in the presence/absence of PKA/CREB1 *in vitro*. The results demonstrated that the ATPase activity of nsp13 was potentiated by CREB1 in a dose-dependent manner (Fig. 3a). In contrast, no such activation was observed after PRKACA treatment (Fig. 3b). Furthermore, by a Förster resonance energy transfer (FRET)-based helicase assay, the helicase activity of nsp13 was also demonstrated to be potentiated by CREB1 (Fig. 3c). For the nsp13 Δ2A mutant (containing only aa 1-442) that failed to bind CREB1, no helicase activity was observed regardless of the presence or absence of CREB1 (Fig. 3d). These results collectively supported that CREB1 regulates nsp13 activity.

To further address the possible role of PRKACA in nsp13 activation, we examined whether phosphorylation of CREB1 at S133 by PRKACA can promote the helicase activity of nsp13 (24). Myc-CREB1 was coexpressed with or without Flag-PRKACA in 293T cells, and the CREB1 protein was purified by affinity purification. As shown in Fig. 3e, CREB1 coexpressed with PRKACA demonstrated a much higher level of CREB1 S133 phosphorylation than CREB1 expressed alone. When coexpressed with PRKACA, CRBE1 showed a significantly stronger potentiation effect on nsp13 helicase activity than CREB1 expressed alone (Fig. 3f), which suggested that PRKACA might contribute to SARS-CoV-2 helicase activation by CREB1 phosphorylation.

### SARS-CoV-2 replication is restrained by PKA-CREB1 inhibition

**Fig 4:**
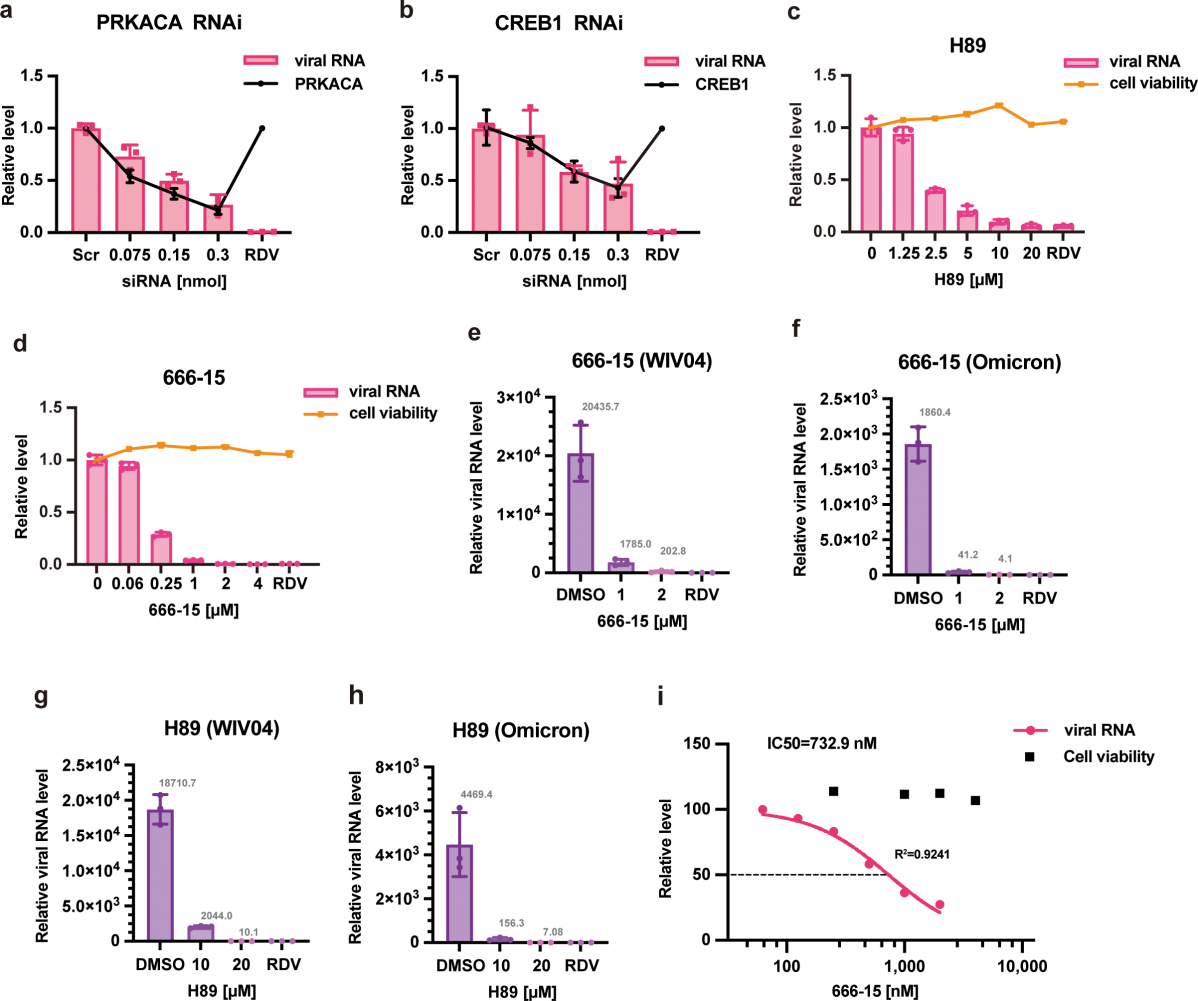
Host CREB1 potentiates SARS-CoV-2 proliferation. (**a, **b) H1299-ACE2 cells were transfected with PKA/CREB1 siRNA or scrambled siRNA at the indicated concentration for 12 h. Then, the cells were infected with trVLPs and Ad-N for 48 h. The effect of the siRNAs on PKA/CREB1 expression (line) and the amounts of trVLPs (column) were determined using quantitative reverse transcription PCR (qRT-PCR). Remdesivir (RDV) (10 µM) was used as a control. (**c, d)** H1299-ACE2 cells were pretreated with H89 or 666-15 at the indicated concentration for 24 h. Then, the cells were infected with trVLPs and Ad-N for 48 h, and the inhibitor was added again at the time of infection. Viral replications were determined using qRT-PCR (column). RDV (10 µM) was used as a positive control. The cytotoxicity of H89 and 666-15 was determined by a CCK-8 assay (line). DMSO and 10 µM RDV were used as controls. (**e–h)** H1299-ACE2 cells infected with live SARS-CoV-2 (WIV04/Omicron strain) at a MOI of 1 were treated with H89 or 666-15 as indicated. DMSO and RDV were used as controls. (**i)** A549-ACE2 cells infected with the live SARS-CoV-2 WIV04 strain at a MOI of 1 were treated with 666-15 as indicated. The percent inhibition of SARS-CoV-2 replication by 666-15 in the A549-ACE2 cell line was determined using qRT-PCR.

Given that host CREB1 upregulates the helicase activity of nsp13, CREB1 may be involved in SARS-CoV-2 replication. To address this issue, viral replication was assessed using a transcription and replication-competent SARS-CoV-2-ΔN virus-like particle (trVLP) system, which could be studied in a biosafety level 2 (BSL-2) laboratory. A complete life cycle of trVLPs with N deletion can be achieved by ectopically expressing complementary SARS-CoV-2 N protein (25). H1299 cells stably expressing the human ACE2 receptor (H1299-ACE2 cells) were used for SARS-CoV-2 trVLP infection and replication. H1299-ACE2 cells were infected with SARS-CoV-2-ΔN trVLPs together with N protein expressing adenovirus Ad-N. Significant viral proliferation was observed upon Ad-N infection, which was suppressed by either PKA- or CREB1-specific RNA interference in a small interfering RNAs (siRNA) dose-dependent manner. The viral replication level was positively correlated with the transcription levels of PKA and CREB1 (Fig. 4a and b).

The role of PKA and CREB1 in SARS-CoV-2 trVLP replication was further assessed by PKA or CREB1 inhibitor treatment. Consistent with the above results, the replication of trVLPs was significantly suppressed by treatment with the PKA inhibitor H89 (Fig. 4c). Compared to vehicle DMSO, trVLP replication was reduced by nearly 150-fold when the cells were treated with 2 µM CREB1 inhibitor 666-15 (Fig. 4d), which was comparable to 10 µM remdesivir, an FDA-approved anti-SARS-CoV-2 drug with an IC50 of 0.77 µM (26).

The effects of PKA/CREB1 on viral replication were further evaluated in live SARS-CoV-2-infected (WIV04/Omicron) H1299-ACE2 cells. Viral proliferation was reduced by ~400-fold with 2 µM 666-15 treatment and up to a 1,800-fold decrease with 20 µM H89 treatment (Fig. 4 through h). Notably, the inhibitors H89 and 666-15 showed similar inhibitory effects on WIV04 and Omicron strain infection (Fig. 4e through h). To evaluate the inhibitory efficiency of 666-15, the IC50 value of 666-15 was determined. The results showed that the IC50 of 666-15 was approximately 732.9 nM, which is comparable to that of remdesivir (Fig. 4i).

## DISCUSSION

The outbreak of SARS-CoV-2 infections has resulted in a global pandemic, with devastating effects on human health and the economy. In this study, we explored the potential mechanisms of the interaction between host PKA-CREB1 and SARS-CoV-2 nsp13 and evaluated the inhibitory effect of PKA/CREB1 inhibitor treatment on SARS-CoV-2 proliferation.

Nsp13 was found to be physically associated with host PKA and CREB1, and this association resulted in enhanced helicase and ATPase activities of nsp13. This enhancement promotes the unwinding of double-stranded helices, thereby promoting SARS-CoV-2 replication. Nsp13 interacts directly with the host protein CREB1, and we believe that CREB1 plays a major role via its interaction with nsp13. Meanwhile, PKA Cα-mediated phosphorylation of CREB1 significantly enhanced its promoting effect on nsp13 helicase activity, which indicated that the upstream kinase PKA also played a role in the regulation of nsp13 activity.

We used protein-protein docking analysis and molecular biology experiments to show that there is a direct interaction between CREB1 and the 2A domain of nsp13. It has also been reported that the 1A and 2A domains of nsp13 coordinate with each other and are involved in nucleic acid binding as well as ATP hydrolysis (7). Notably, nsp13 Arg442 and Lys569 were also shown to be able to interact with the nsp13 inhibitor licoflavone C (27), and Arg507 and Lys508 are situated on the surface of the 2A domain and exposed to solution; mutations of these residues can result in instability of the complete structure of SARS-CoV-2 nsp13 (7). We speculated that interactions with these residues may affect the helicase activity of nsp13 by affecting its structural stability, which may increase the binding strength of nsp13 to nucleic acids, thereby increasing its helicase activity. Since our experiment showed that the ATPase activity of nsp13 was also enhanced upon the addition of CREB1, the rate of ATP utilization by nsp13 may be enhanced, in turn enhancing its helicase activity. However, further investigations are needed to determine the exact mechanism by which CREB1 facilitates SARS-CoV-2 proliferation.

**Fig 5:**
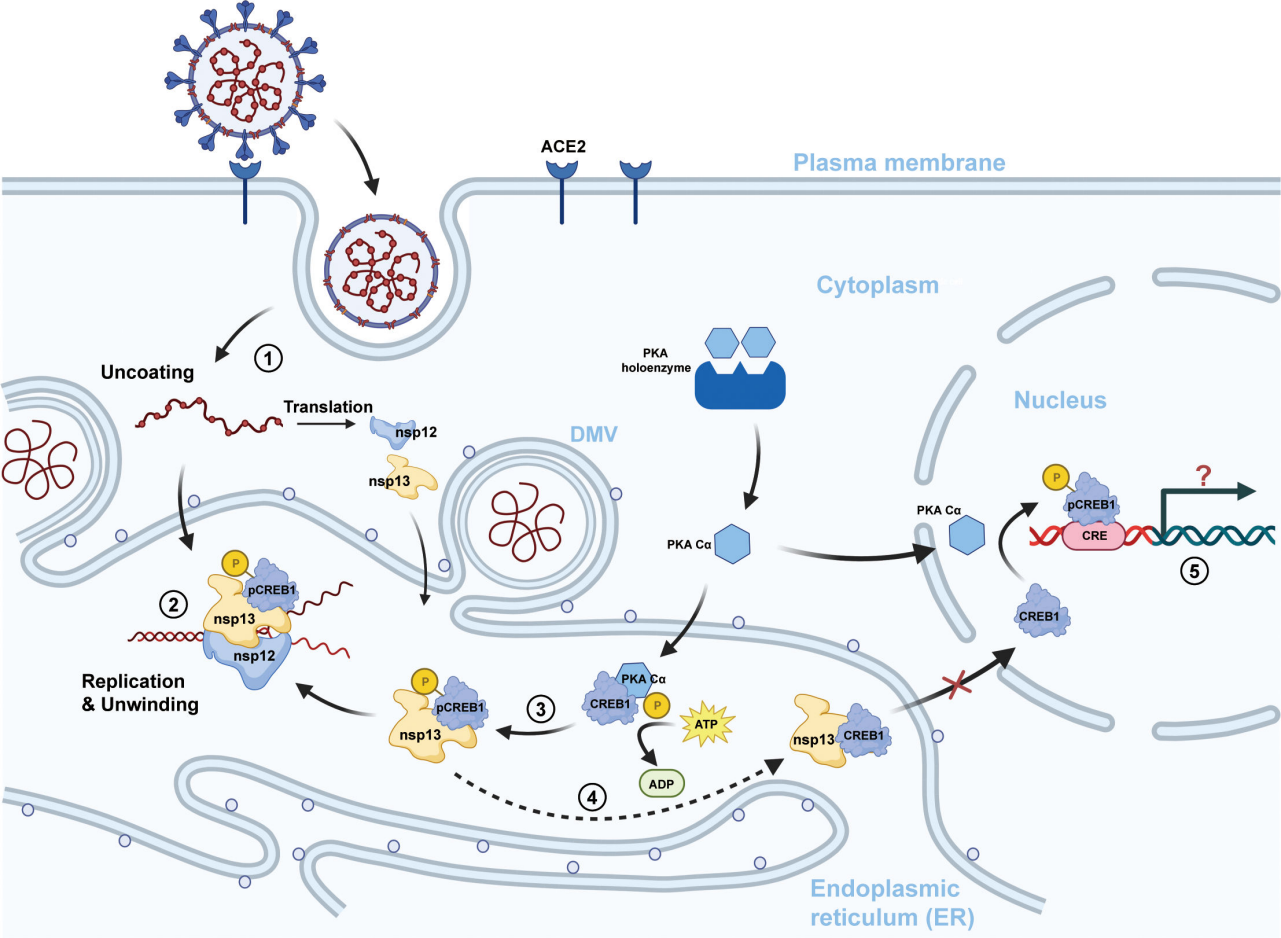
Graphical view of the mechanism by which the PKA-CREB1 axis regulated viral replication through the nsp13:CREB1 interaction. (1) Following entry and uncoating, the RNA genome of SARS-CoV-2 is translated to generate a viral replication complex from ORF1a/b (including nsp12 and nsp13). (2) The helicase nsp13 directly interacts with the host transcription factor CREB1 at the site of viral replication and transcription, resulting in enhanced helicase and ATPase activities of nsp13. (3) PKA Cα-mediated phosphorylation of CREB1 enhanced its promoting effect on nsp13 helicase activity. (4) Viral nsp13 binds CREB1, resulting in the accumulation of CREB1 in the cytoplasm and preventing it from entering the nucleus. (5) Whether the reduction of CREB1 in the nucleus will affect the life activities of the host and its relationship with the pathogenic mechanism of the virus remains to be studied.

The subcellular localization of the interaction was investigated by immunofluorescence, and nsp13 was observed to be colocalized with the PKA-CREB1 complex in the endoplasmic reticulum region of the cell, and colocalization was not observed in the Golgi apparatus (data not shown). Therefore, we speculated that CREB1 is hijacked by nsp13 and transported into the bilayer membrane structure formed by the endoplasmic reticulum, which is responsible for SARS-CoV-2 transcription and replication (Fig. 5). Accumulation of CREB1 in the cytoplasm but not the nucleus was observed upon viral infection (Fig. 1d), which suggested that SARS-CoV/SARS-CoV-2 infection may attenuate the expression of CREB1 downstream genes, which play important roles in cell growth, immune regulation, and gluconeogenesis, and that the nsp13/CREB1 interaction may play potential roles in the pathogenesis of the virus.

Targeting viral proteins has historically been a common strategy for the development of antiviral drugs. For example, remdesivir, which targets RdRp (nsp12), has been shown to reduce viral replication and improve patient outcomes in clinical trials (28). However, rapid mutation in the viral genome (particularly in the presence of antiviral agents) may lead to the emergence of drug-resistant strains. Agents targeting host proteins may have broader-spectrum activity against diverse viral strains that employ the same pathway of the host for proliferation and pathogenesis. In this work, understanding the interaction between host CREB1 and SARS-CoV-2 nsp13 may have significant implications for the development of therapeutics against COVID-19. The CREB1 inhibitor and PKA inhibitor showed promising efficacy in suppressing viral replication in SARS-CoV-2-infected cells (Fig. 4e through i). Knocking down PKA and CREB1 expression also exerted a similar inhibitory effect on virus proliferation (Fig. 4a and b).

The finding that the PKA-CREB1 signaling pathway is hijacked by nsp13 to facilitate SARS-CoV-2 replication provides new potential therapeutic targets for COVID-19 and related diseases. Notably, the CREB1 inhibitor 666-15 showed no significant difference in the proliferation of different strains of SARS-CoV-2 (Fig. 4e through h). And in the WIV04 strain infection, the inhibitory effect of 666-15 on virus proliferation is similar to that of remdesivir, with an IC50 of 732.9 nM (Fig. 4i). Considering that there was only one amino acid difference between nsp13 of SARS-CoV and SARS-CoV-2, PKA/CREB1 inhibition may provide an approach for the development of broad-spectrum anti-SARS-CoV/SARS-CoV-2 therapeutics.

## MATERIALS AND METHODS

### Cell lines and transfections

293T, H1299, and Caco-2 cells were grown in Dulbecco’s modified Eagle’s medium (DMEM, Gibco). A549 cells were grown in Kaighn’s Modification of Ham’s F-12 medium (F-12K, Gibco). All media were supplemented with 10% fetal bovine serum (Gibco), 100 units/mL penicillin, and 100 µg/mL streptomycin, and cells were grown at 37°C under an atmosphere of 5% CO_2_. The H1299 cells used for trVLP or live SARS-CoV-2 infection studies were infected with lenti-ACE2, and stable clones were selected by screening with puromycin (1 µg/mL). Cells were treated with H89 (Selleck), 666-15 (Selleck), and remdesivir (Selleck). The cytotoxicity of the drugs used in this study was tested by a CCK-8 assay (Solarbio). Transient transfection was performed with Lipofectamine 2000, Lipofectamine 3000 (Invitrogen), or the TransIT-X2 dynamic delivery system (Mirus) according to the manufacturer’s instructions.

### Mice

Specific pathogen-free BALB/c mice were purchased from Beijing Vital River Laboratory Animal Technology Co., Ltd. Six- to eight-weeks-old (18 to 22 g) mice were used in the experiments. All animal studies were completed in the Animal Care Facilities of Changchun Veterinary Research Institute.

### Vectors and viruses

The Flag-tagged PRKACA (catalytic subunit of PKA) expression plasmid was constructed by inserting the PRKACA gene fragment into a pcDNA3.0-based Flag vector (Invitrogen). The Flag-tagged nsp13 expression plasmid was constructed by inserting the nsp13 gene fragment into the pCAGGs vector (Clontech). The Myc-tagged PRKACA and CREB1 expression plasmids were constructed by inserting the corresponding gene fragments into the pCMV-Myc vector (Clontech). GFP-tagged nsp13 expression plasmids were constructed by inserting the nsp13 gene fragment into pEGFP-C1 (Clontech). The GST-tagged nsp13 and nsp13 deletion and truncation mutant expression plasmids were constructed by inserting the corresponding gene fragments into the pGEX-4T-1 vector (Promega). All the constructs were validated by Sanger sequencing. SARS-CoV-2 (WIV04/Omicron) and mouse-adapted SARS-CoV-2 (C57MA14) have been described previously (29). The trVLPs were produced according to a previously reported description (25).

### Immunoprecipitation and immunoblot analysis

Cell lysates were prepared in prechilled Mammalian Protein Extraction Reagent (Thermo Scientific) containing protease inhibitor cocktail (Roche). Soluble proteins were immunoprecipitated using anti-Flag (Millipore, A2220) and anti-GFP (ABclonal, AE074) agarose beads or species-matched IgG of the same isotype as a negative control (Sigma-Aldrich, A0919 or A2909). An aliquot of the total lysate (5%, vol/vol) was included as a control. Immunoblotting was performed with horseradish peroxidase (HRP)-conjugated anti-Flag (Sigma, A8592, 1:2,000 dilution), HRP-conjugated anti-Myc (Sigma, SAB4200742, 1:2,000 dilution), HRP-conjugated anti-GFP (Santa Cruz, sc-8334, 1:500 dilution), HRP-conjugated anti-GST (Invitrogen, MA4-004-HRP, 1:1,000 dilution), anti-PRKACA (BD Biosciences, 610980, 1:1,000 dilution), anti-CREB1 (Proteintech, 12208-1-AP, 1:1,000 dilution), and anti-Phospho-CREB (Ser133) antibodies. The antigen-antibody complexes were detected using an enhanced chemiluminescence system (GE Healthcare) and Immobilon Western HRP Substrate (Millipore, WBKLS0100). PageRuler Prestained Protein Ladder (Thermo) was used as a molecular weight standard.

### SARS-CoV-2 nsp13 expression and purification

The full-length SARS-CoV-2 nsp13 helicase and its mutants were expressed as GST-tagged fusion proteins in *E. coli* BL21 (DE3); GST was fused to the N-terminus of nsp13. Cells were grown at 37°C, and protein expression was induced with 0.5 mM isopropyl-D-thiogalactopyranoside when the OD_550_ was 0.6–0.8 and was continued during incubation for 16 h at 18°C. Cells were harvested and resuspended in PBST containing a protease inhibitor cocktail. Suspended cells were sonicated and centrifuged at 25,000 *g* at 4°C for 30 min. Then, the GST-nsp13 and mutant proteins were purified using Glutathione Sepharose 4B Affinity media (Cytiva) and eluted with buffer (50 mM Tris-HCl, 10 mM reduced glutathione, pH 8.0). The eluate was then further purified on a PD SpinTrap G-25 buffer exchange column with TBS buffer (Cytiva). The purity of the proteins was determined by Coomassie blue staining.

### GST pulldown, far western blotting, and dot blotting assay

Lysates of cells transfected with the indicated overexpression plasmids were incubated with purified GST or GST fusion proteins conjugated to glutathione beads. The precipitates were then washed with lysis buffer and subjected to SDS-PAGE and immunoblot analysis. An aliquot of the total lysate (5%, vol/vol) was included as a control.

Far western blotting was conducted in the direct binding assay. Purified GST or the GST-nsp13 fusion and its mutants were separated by SDS-PAGE and transferred onto PVDF membranes. The membranes were subsequently incubated with purified CREB1 or PRKACA protein (Origene) for 1 h at room temperature. The direct binding of the GST fusion proteins was detected using an anti-CREB1 or anti-PRKACA antibody. The purity of the proteins was determined by Coomassie blue staining.

In the dot blotting assay, the concentration of purified GST, GST fused full-length nsp13, and its mutants were determined by the Pierce Rapid Gold BCA Protein Assay Kit (Thermo Scientific). Then spot 2 µL (3 µg) of each protein onto the nitrocellulose membrane and place the spotted membrane in a constant temperature incubator at 37°C for 30 min. After blocking, the membrane was incubated with purified CREB1 or PRKACA protein (Origene) for 1 h at room temperature. The direct binding of the GST fusion proteins was detected using an anti-CREB1 or anti-PRKACA antibody. The purity of the proteins was determined by Coomassie blue staining.

### Immunofluorescence microscopy

A549 and Caco-2 cells were transfected or infected as described above. Cells on glass coverslips were fixed with paraformaldehyde (PFA; 4%) for 10 min and were then permeabilized with 0.2% Triton X-100 in PBS for 15 min. After blocking with 0.2% goat serum in PBS for 30 min, the cells were incubated with rabbit anti-nsp13 (ABclonal, A20311, 1:50 dilution), mouse anti-PRKACA (BD Biosciences, 610980, 1:100 dilution), or mouse anti-CREB (Abcam, ab178322, 1:100 dilution) antibodies overnight at 4°C. The cells were washed three times with PBS and then incubated with FITC- or TRITC-conjugated goat anti-rabbit or anti-mouse IgG. The cells were then washed, stained with DAPI, and imaged using a laser scanning confocal microscope (Zeiss LSM 800 Meta, built-in software ZEN2.3) with a 63× oil immersion objective lens. The signal intensity was analyzed by Fiji (ImageJ) software (30).

### SARS-CoV-2 trVLP assay

With reference to the publication by Ding et al. (25), a BSL-2 cell culture system for the production of trVLPs was established. These trVLPs express all viral proteins except the N protein, which is required for viral proliferation. The viral life cycle can be completed in cells by ectopically expressing the SARS-CoV-2 N protein. In brief, H1299-ACE2 cells were infected with Ad-N and SARS-CoV-2-ΔN trVLPs. One day after infection, the medium was replaced, and inhibitors were added at the indicated concentration. After incubation for another 3 days, the cells were harvested, and viral RNA titers were determined by reverse transcription and quantitative reverse transcription PCR (qRT-PCR).

### *In situ* PLA

The associations of SARS-CoV-2 nsp13 with PRKACA and CREB1 in lung sections from infected mice and COVID-19 patients were investigated with *in situ* Proximity Ligation Assay Reagent (Sigma-Aldrich, DUO92008). In brief, after sections were sliced, the sections on glass slides were fixed, permeabilized, and blocked as described above. Then, the sections were incubated with rabbit anti-nsp13 (ABclonal, A20311, 1:50 dilution), mouse anti-PRKACA (BD Biosciences, 610980, 1:100 dilution), or mouse anti-CREB (Abcam, ab178322, 1:100 dilution) primary antibodies. The anti-nsp13 antibody alone was employed as a negative control. After washing three times with PBS, the sections were incubated with affinity-purified donkey anti-rabbit or anti-mouse IgG (Sigma-Aldrich, DUO92002, DUO92004) secondary antibodies. The red fluorescent puncta were generated via a DNA amplification-based reporter system, and nuclei were stained with DAPI (blue). Images were acquired using a laser scanning confocal microscope (Zeiss LSM 800 Meta, built-in software ZEN2.3) with a 63× oil immersion objective lens.

### SARS-CoV-2 nsp13 ATPase assay

The ATPase assay was performed using an ATPase/GTPase Activity Assay Kit (Sigma-Aldrich, MAK113). The reactions were performed in clear flat-bottom 96-well plates (Corning) and consisted of 40 µL of reaction mix containing 500 nM purified GST-nsp13 and 1 mM ATP. PRKACA (Origene, TP310332) or CREB1 (Origene, TP760318) was added to the reaction mix at the indicated concentration. After 30 min of preincubation at room temperature, 200 µL of reaction reagent was added, and the plates were incubated for 30 min at room temperature. Reaction products were quantified by measuring the absorbance at 620 nm.

### FRET-based SARS-CoV-2 nsp13 helicase assay

A FRET-based fluorescence-quenching approach was used to monitor nucleic acid duplex unwinding catalyzed by nsp13. Since there was no preference for either dsRNA or dsDNA (31), our experimental analysis was carried out using dsDNA substrates, in which one strand of the duplex was labeled with Cy3, while the other was labeled with Black Hole Quencher (5′-AGT CTT CTC CTG GTG CTC GAA CAG ACG C-Cy3-3′, 5′-BHQ-2-GCG TCT GTT CGA GCA CCA CCT CTT CTG A-3′). Moreover, an ssDNA bearing a sequence complementary to the Cy3-labeled strand but without modification was added to the reaction system to capture the Cy3-labeled strand that was released upon duplex unwinding (5′-GCG TCT GTT CGA GCA CCA-3′). HPLC-purified DNA oligonucleotides were purchased from Tsingke Biotechnology. The Cy3-labeled strand and the quenched strand were annealed at a 1:1.2 ratio by heating the oligo mix to 95°C for 5 min and gradually cooling it to 25°C over a period of 90 min in reaction buffer (10 mM Tris-HCl pH 7.5–8.0, 50 mM NaCl). The unwinding activity was measured in black 384-well plates (PerkinElmer) in a 40 µL reaction mix containing 50 mM HEPES pH 7.5, 20 mM NaCl, 4 mM MgCl_2_, 1 mg/mL BSA, 1.2 µM competitor strand, and 100 nM purified nsp13. The reaction mix containing the enzyme was preincubated for 15 min with PRKACA (Origene, TP310332) or CREB1 (Origene, TP760318) at room temperature. The reaction was started by adding 2 mM ATP and 200 nM dsDNA substrate. After a 15-min incubation at 37°C, the fluorescence signal was monitored at an excitation wavelength of 550 nm and an emission wavelength of 570 nm with a 5 nm bandwidth using an EnVision Multilabel Plate Reader (PerkinElmer).

To investigate the effect of PRKACA-mediated CREB1 phosphorylation on nsp13 activation, Myc-CREB1 and s133-phosphorylated Myc-CREB1 were prepared from 293T cells expressing Myc-CREB1 and Flag-PRKACA or Flag-vector by affinity purification with anti-Myc conjugated agarose beads. The phosphorylation level of purified Myc-CREB1 was analyzed by immunoblot with anti-Phospho-CREB (Ser133) antibody. Purified Myc-CREB1 was added to the reaction mix at the indicated concentration and the experiment was conducted as described before.

### Reverse transcription and qRT-PCR

Total cellular RNA was extracted from cells (~10^5^ cells) using the RNeasy Plus Mini Kit (QIAGEN, 74134) or QIAamp Viral RNA Mini Kit (QIAGEN, 52904) according to the manufacturer’s protocol. For cDNA synthesis, RNA samples were reverse transcribed using the GoScript Reverse Transcription System (Promega, A5001) in a 20 µL volume. Then, 1 µL of cDNA was used as a template for qPCR. The sequences of the primers used are shown in Table S1. qRT-PCR was performed using GoTaq qPCR Master Mix (Promega, A6001) with a QuantStudio 6 Flex multicolor PCR system (ABI). GAPDH was used as a control for the normalization of RNA levels, and relative RNA expression was calculated using the 2^-ΔΔCt^ method. Viral RNA was quantified by targeting NP as described previously (32). The mean ± SEM (error bars) of three independent experiments are presented in the figures.

### Docking of CREB1 on the SARS-CoV-2 Nsp13 structure

Protein-protein docking analysis of the binding of CREB1 to SARS-CoV-2 nsp13 was conducted using the ClusPro 2.0 web server (22, 23). In brief, the starting structure of nsp13 was obtained from the Protein Data Bank (www.rcsb.org) (PDB ID: 7NIO), and the three-dimensional structure of CREB1 was predicted with AlphaFold. The docking results were ranked by the parameters of van der Waals and electrostatic interactions, and the best result, which had the lowest binding energy, was selected for further analysis.

### Gene silencing using siRNA

For gene knockdown in H1299-ACE2 cells, cells cultured in six-well plates were transfected with 100 pmol of PRKACA siRNA (sense, 5′- CCA GAU CGU CCU GAC CUU UTT-3′; antisense, 5′- AAA GGU CAG GAC GAU CUG GTT-3′), 100 pmol of CREB1 siRNA (sense, 5′- GGC CUG CAA ACA UUA ACC ATT-3′; antisense, 5′- UGG UUA AUG UUU GCA GGC CTT-3′), or 100 pmol of scrambled siRNA (sense, 5′-UUC UCC GAA CGU GUC ACG UTT-3′; antisense, 5′-ACG UGA CAC GUU CGG AGA ATT-3′), purchased from Tsingke Biotechnology, with the TransIT-X2 delivery system (Mirus) according to the manufacturer’s recommendations.

### Animal experiments

Infection of BALB/c mice was performed as described previously (29). Six- to eight-week-old BALB/c mice (three per group, female, 6–8 weeks old) were purchased from Beijing Vital River Laboratory Animal Technology Co., Ltd. The use of animals complied with all relevant ethical regulations and was approved by the committee of the Laboratory Animal Center, Changchun Veterinary Research Institute. In the infection group, each BALB/c mouse was inoculated intranasally with 1 × LD50 (10^3^ TCID50) mouse-adapted SARS-CoV-2 (C57MA14). Mice in the control group were treated with the same dose of PBS. Mice from each group were euthanized at 3 dpi, and lung tissues were collected for immunofluorescence staining.

### Statistical analysis

Graphical representations were generated and statistical analyses were performed using Prism 8 software. The results are presented as the mean (upper limit of the box) ± SEM (error bars) of three or more independent experiments, and significance was analyzed by Student’s *t*-test. Colocalization analysis was performed using Fiji software. Differences with *P* values of <0.05 (*), <0.01 (**), and <0.001 (***) were considered significant.

## SUPPLEMENTAL MATERIAL

Supplemental material jvi.01565-23-s0001.docx Figures S1 and S2; Table S1.

ASM does not own the copyrights to Supplemental Material that may be linked to, or accessed through, an article. The authors have granted ASM a non-exclusive, world-wide license to publish the Supplemental Material files. Please contact the corresponding author directly for reuse.
