# Supplementary material for: The PKA-CREB1 axis regulates coronavirus proliferation by viral helicase nsp13 association
Source: J Virol. 2024 Mar 6;98(4):e01565-23. doi: 10.1128/jvi.01565-23 (PMC11019953; doi:10.1128/jvi.01565-23)
Supplement: Supplemental material — Figures S1 and S2; Table S1. [file jvi.01565-23-s0001.docx]

**Supplementary Information**

**The PKA-CREB1 Axis Regulates Coronavirus Proliferation by Viral Helicase Nsp13 Association**

Tong Zheng^1^, Beilei Shen^2^, Yu Bai^3^, Entao Li^2,4^, Xun Zhang^3^, Yong Hu^1^, Ting Gao^1^, Qincai Dong^1^, Lin Zhu^1^, Rui Jin^1^, Hui Shi^1^, Hainan Liu^1,*^, Yuwei Gao^2,*^, Xuan Liu^1,*^, and Cheng Cao^1,*^

**Affiliations**

^1^Beijing Institute of Biotechnology, Beijing 100850, China.

^2^Changchun Veterinary Research Institute, Chinese Academy of Agricultural Sciences, Changchun 130000, China.

^3^Institute of Physical Science and Information Technology, Anhui University, Hefei, Anhui 230601, China.

^4^Division of Life Sciences and Medicine, University of Science and Technology of China, Hefei, Anhui 230026,China.

*Correspondence to:

Cheng Cao (Phone: +86-10-66948824; E-mail: caoc@nic.bmi.ac.cn)

Xuan Liu (Phone: +86-10-66948824; E-mail: liux931932@163.com)

Yuwei Gao (Phone: +86-0431-86985801; E-mail: yuwei0901@outlook.com)

Hainan Liu (Phone: +86-10-66948824; E-mail: Jerrylhn@126.com)

**
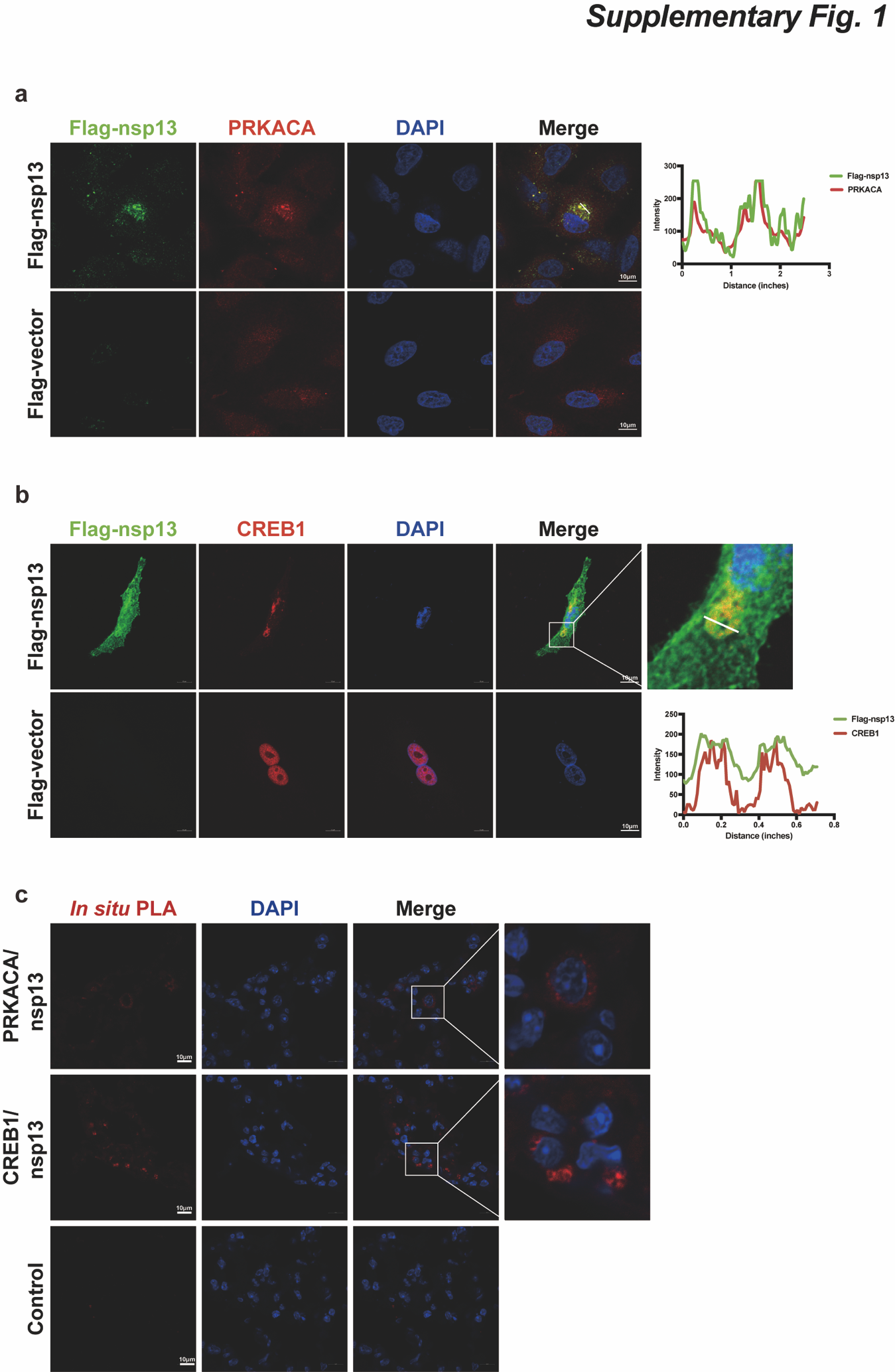
**

**Supplementary Fig. 1 nsp13 colocalization with host PRKACA and CREB1.**

**a, b** A549 cells were transfected with the Flag-nsp13 plasmid and were then subjected to immunostaining with anti-PRKACA (a) or anti-CREB1 (b) and anti-nsp13 antibodies. The fluorescence intensity was analyzed by Fiji (ImageJ) software. c, Lung tissue sections of mice infected with a mouse-adapted strain of SARS-CoV-2 were subjected to an in situ PLA with anti-nsp13 and anti-PRKACA or anti-CREB1 antibodies.


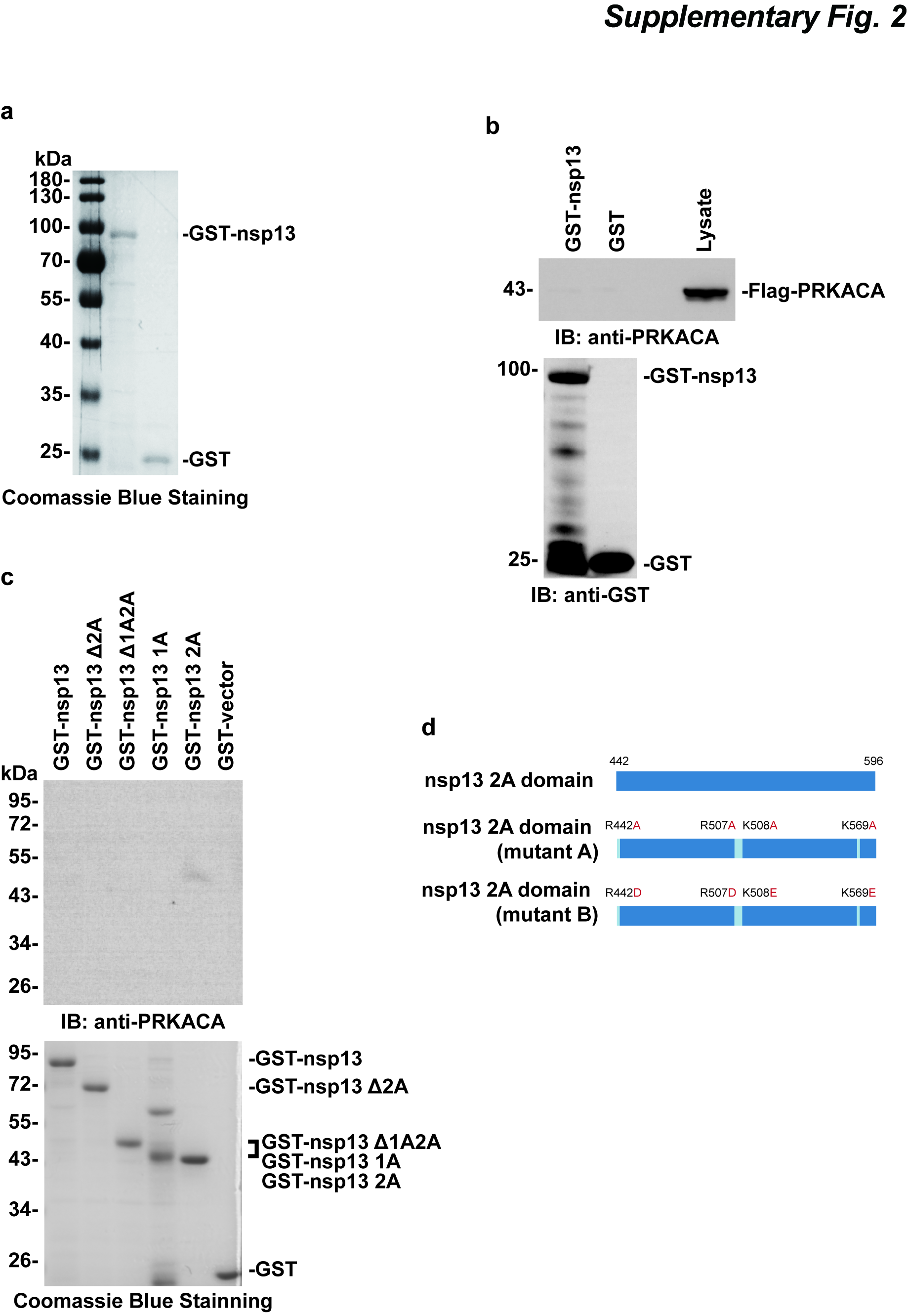


**Supplementary Fig. 2 Direct interaction between nsp13 and host PKA.**

**a,** Purified GST-nsp13 and GST were analyzed by SDS-PAGE and Coomassie blue staining. **b,** The interaction of SARS-CoV-2 nsp13 with PRKACA was evaluated by a GST pulldown assay. Lysates of HEK293T cells transfected with the Flag-PRKACA plasmid were incubated with GST-nsp13 and GST agarose beads. Precipitates and lysates were analyzed by immunoblotting, and GST protein was used as a negative control. **c,** The far western assay of nsp13 and PRKACA interaction was conducted as a negative control. Purified GST-nsp13 and its mutants (described in Fig. 2b) were resolved by SDS-PAGE and transferred onto a PVDF membrane. The PVDF membrane was incubated with purified PRKACA and then subjected to immunoblotting with an anti-PRKACA antibody. The purity of GST-nsp13 and its mutants were analyzed by Coomassie blue staining. **d**, Schematic diagrams of 2A domain (aa442 to 596) point mutants in nsp13. R442A, R507A, K508A and K569A mutations is represented by mutant A. R442D, R507D, K508E and K569E mutations is represented by mutant B.

**Supplementary Table 1. Primers used for qRT-PCR.**

| Gene Product | Forward primer (5’ to 3’) | Reverse primer (5’ to 3’) |
| --- | --- | --- |
| h-GAPDH | GGAGCGAGATCCCTCCAAAAT | GGCTGTTGTCATACTTCTCATGG |
| h-PRKACA | AGCCCACTTGGATCAGTTTGA | GTTCCCGGTCTCCTTGTGT |
| h-CREB1 | ATTCACAGGAGTCAGTGGATAGT | CACCGTTACAGTGGTGATGG |
